# Supplementary material for: A High Sensitivity Self-Powered Wind Speed Sensor Based on Triboelectric Nanogenerators (TENGs)
Source: Sensors (Basel). 2021 Apr 23;21(9):2951. doi: 10.3390/s21092951 (PMC8122800; doi:10.3390/s21092951)
Supplement: Supplementary file 1 [file sensors-21-02951-s001.zip › sensors-1174288-supplementary.pdf]

Supporting Information

A high sensitivity self-powered wind speed sensor based on triboelectric nanogenerators (TENGs)

Yangming Liu, Jialin Liu, Lufeng Che*

College of Information Science & Electronic Engineering, Zhejiang University, Hangzhou 310027, China.
* Correspondence：lfche@zju.edu.cn（L. F. Che）

Figure 1. shows a physical image of TENG in the self-powered wind speed sensor, including different lengths, widths, and more.

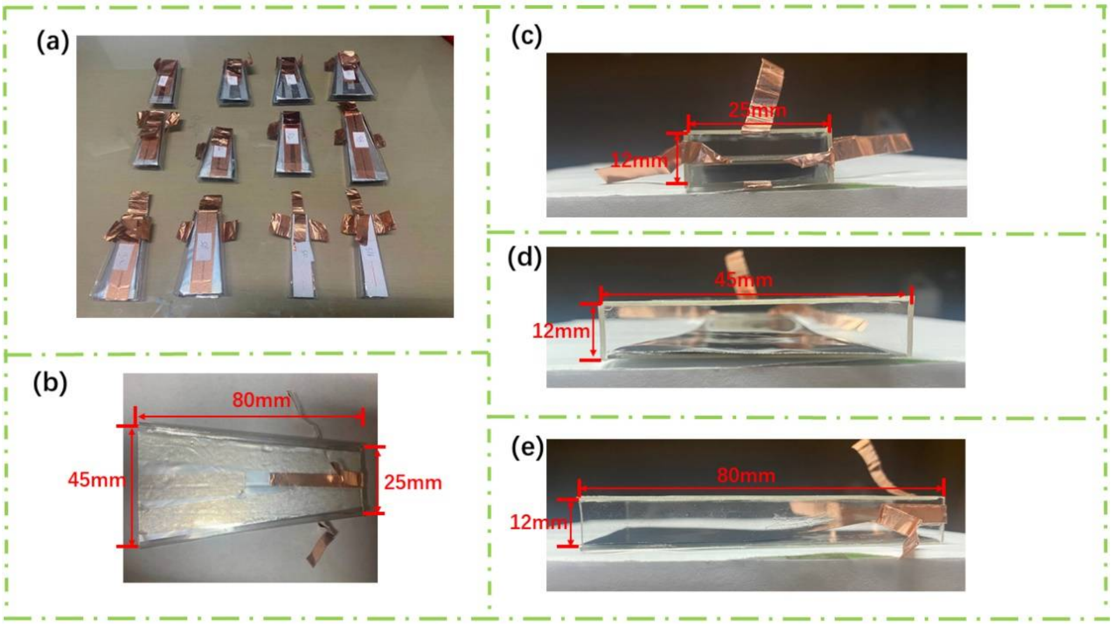

Figure S1. (a)Part of sensors' physical drawings with different geometric parameters. (b)The top view of the sensor. (c) The Air inlet of TENG. (d) The Air exit of the sensor. (e)The side view of the sensor.

Figure S2 shows the schematic diagram for wind direction measurement.

The design of this trapezoidal structure has two advantages: on the one hand, it can increase the contact area between friction layers, so we can obtain more transferred charges and achieve greater output. On the other hand, the trapezoidal design enables the sensor to achieve another function besides wind speed measurement—wind direction measurement. As shown in Figure. S2, it will have a function similar to that of a wind vane when the sensor is placed on a rotatable turntable. According to the Bernoulli principle, the pressure between the two sides of the trapezoid is different, which will rotate the sensor with the external wind. According to the above description, a wind direction measurement can be achieved.

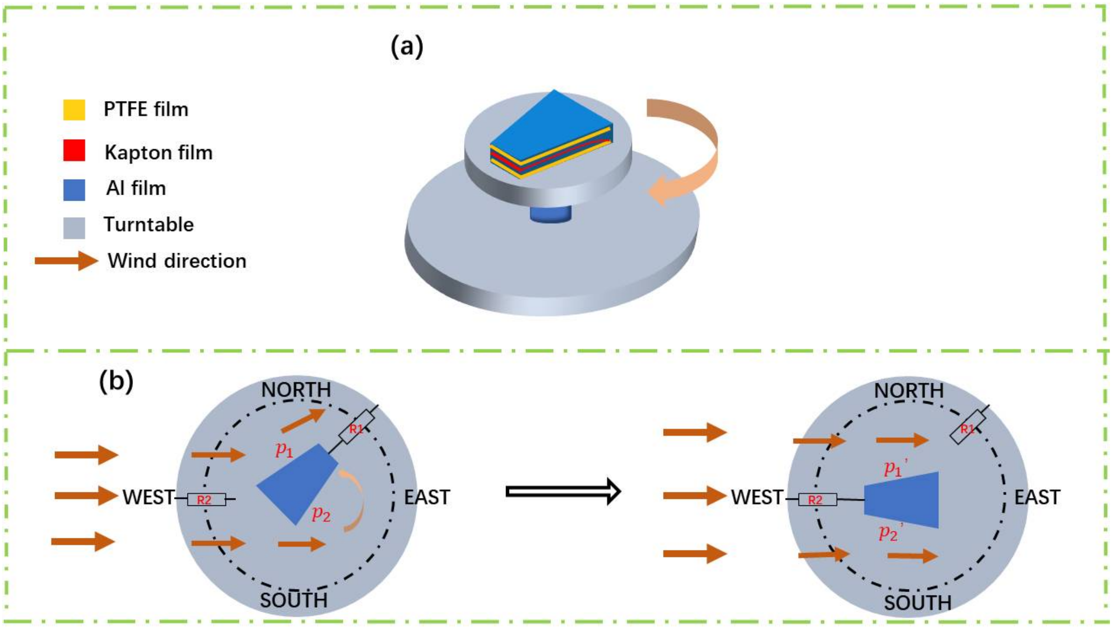

Figure S2. Schematic diagram for wind direction measurement.

Figure S3 shows the side view of the wind speed sensor and the motion of the Al/Kapton/Al film.

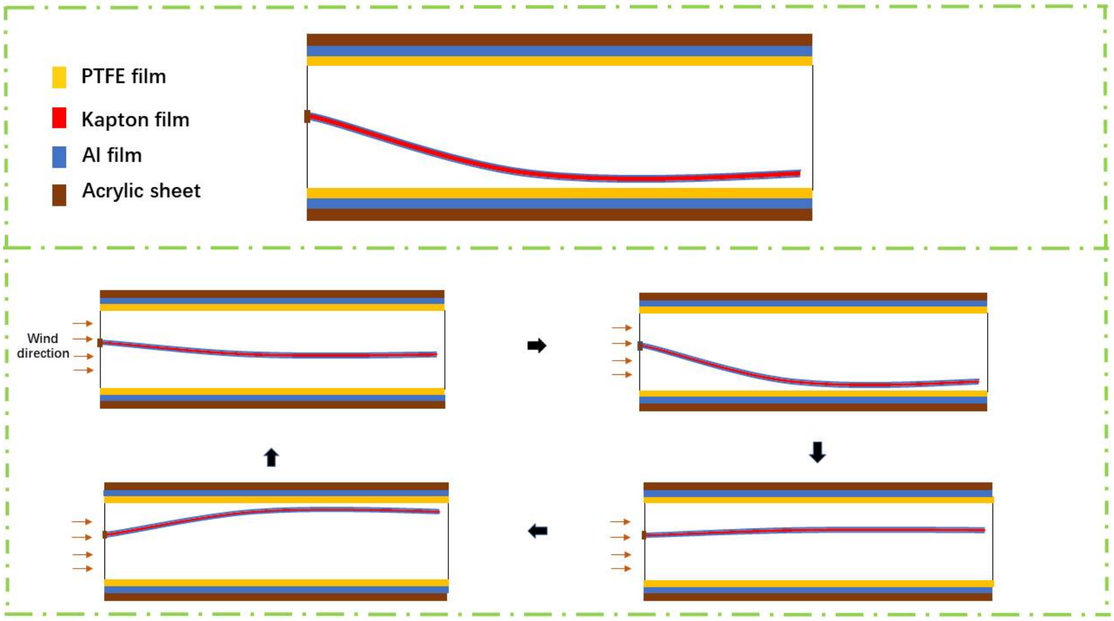

Figure S3. The side view of the wind speed sensor and the motion of the Al/Kapton/Al film.

Figure S4 shows the schematic diagram for the DAQ model.

The wind speed sensor can generate a legible current signal when the Al/Kapton/Al film floats up or down. Consequently, a self-powered wind speed sensor can be constructed. The current signal is directly sent to a Labview platform for processing through a data acquisition (DAQ) module. Then, by calculating the average value of the peak current, the computed output current can be displayed on the screen as shown in Figure S4.

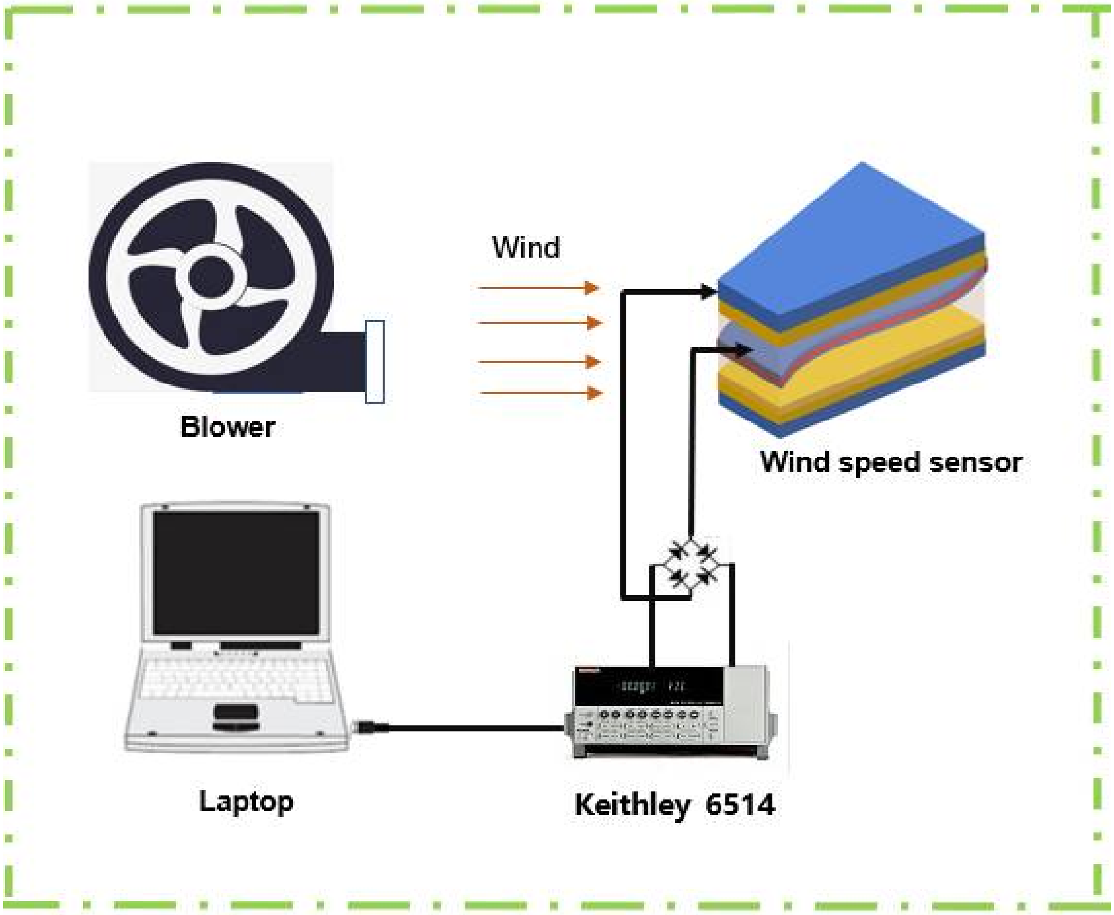

Figure S4. The schematic diagram for DAQ model.

Figure S5 shows the vibration frequency of the wind speed sensor.

In two days, we calculated the time interval between two peak current and obtained the frequency of the output current.

We also measured the change of the frequency of the output current under different wind speed conditions, which is shown in Figure S5(a). The frequency of the output current increases with the increase of wind speed. We measured the frequency to further explain the influence on the current caused by the ratio of the topline and baseline length of the trapezoid.

We also measured the frequency of the output current with different structure parameters. The contact area between the friction layers increases along with the ratio. Hence, we can obtain more transfer charges. However, as shown in Figure S5(c), the increased of the ratio makes the gravity of the Al/Kapton/Al film increase, which will decrease the vibration frequency. Furthermore, the decrease of the vibration frequency will lead to the decrease of its output current. Therefore, the impact of both aspects leads to the trend of output current in the manuscript. The explanation of vibration frequency also applies to the trend in Figure 4(c).

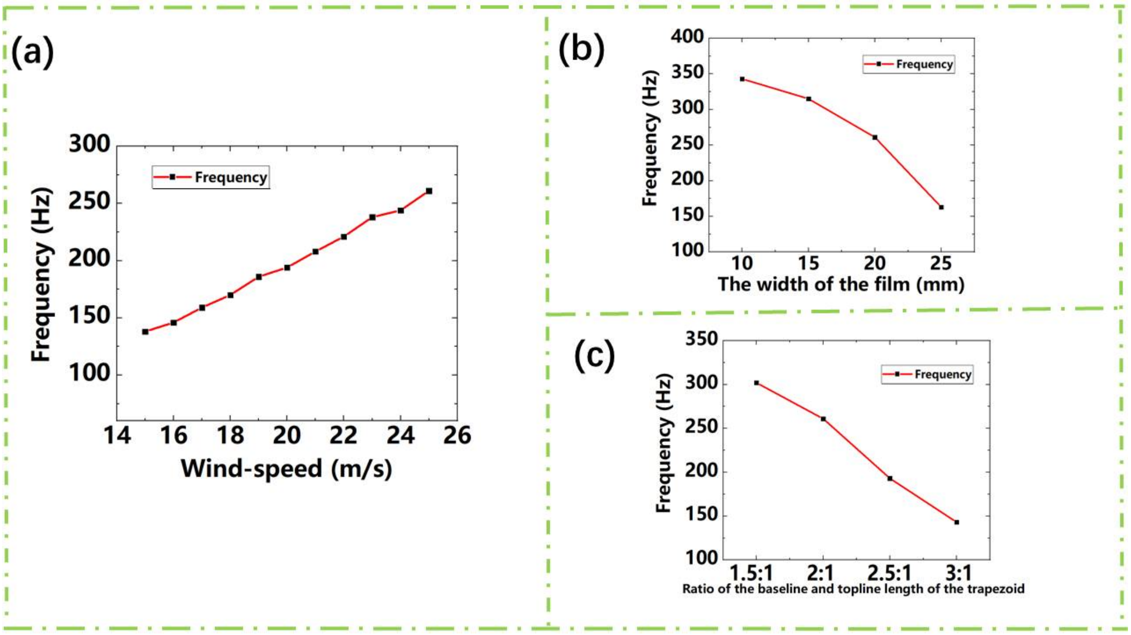

Figure S5. (a) The vibration frequency under different wind speeds. (b) The vibration frequency with different widths of the film. (c) The vibration frequency with different ratios of the topline and baseline length of the trapezoid.

Figure S6 shows the Labview program which can convert the TENG-1 and TENG-2 output into wind speed.

We made a Labview program that can convert the TENG-1 and TENG-2 output into wind speed as shown in Figure S6. The program includes two parts. One of the parts is a peaks acquisition model which can calculate the obtained data and then obtain a computed current value. The other part is a formula calculation model, in which we input a computed current value to get the corresponding wind speed value.

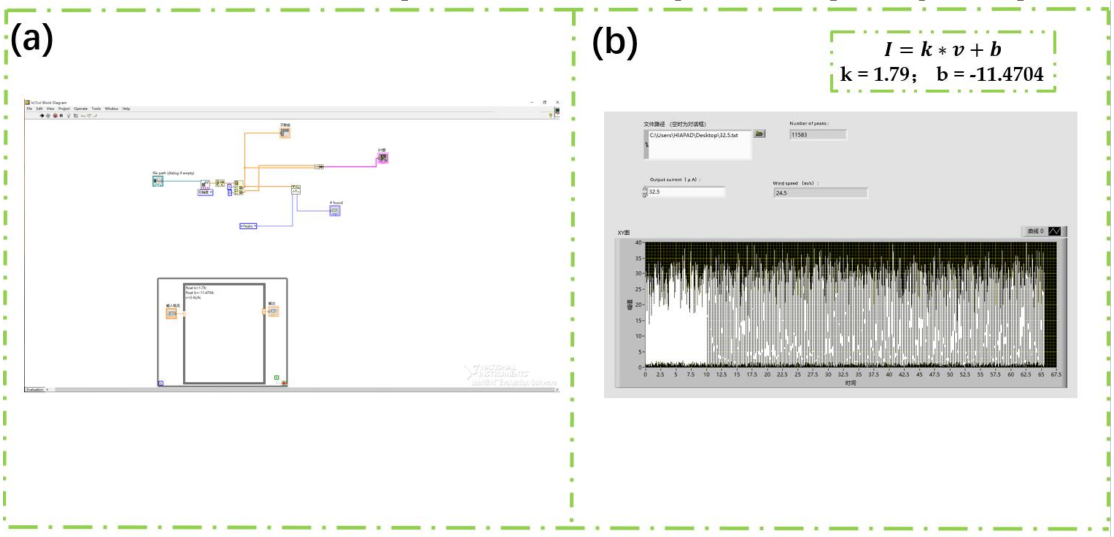

Figure S6. The Labview program with the wind speed of 24.5 m/s.
